# Gastrointestinal Stromal Tumor With a Rare Associated Meningioma: A Case Report

**DOI:** 10.7759/cureus.31361

**Published:** 2022-11-11

**Authors:** Daniel Miller, Asma Hosna, Karim Makhoul, Toka Amin, Daniel Fuchs

**Affiliations:** 1 Internal Medicine, Icahn School of Medicine at Mount Sinai, Queens Hospital Center, New York City, USA; 2 Neurology, Icahn School of Medicine at Mount Sinai, Queens Hospital Center, New York City, USA; 3 Internal Medicine, Cairo University Faculty of Medicine, Cairo, EGY; 4 Internal Medicine, Coney Island Hospital, Brooklyn, USA

**Keywords:** brain tumor, gastrointestinal disease, serum tumor markers, meningioma, gastrointestinal stromal tumor (gist)

## Abstract

Gastrointestinal stromal tumors (GISTs) tend to be associated with other tumors. In certain familial cancer syndromes, GIST has been associated with breast cancer or other endocrine tumors. Multiple endocrine neoplasia syndrome along with certain other genomic mutations such as succinate dehydrogenase complex mutations described GIST as one of the potential tumors of the syndrome. There has not yet been a definite association between GIST and meningioma. We present a case of a patient with a GIST who was later found to have a meningioma on incidental brain imaging. Despite being a benign tumor not requiring additional intervention, it is quite apparent that providers need to have a low threshold to scan for other tumors if suspicious symptoms arise.

## Introduction

A gastrointestinal stromal tumor (GIST) is a rare neoplasm that arises from mesenchymal cells and is found in the gastrointestinal (GI) tract. While metastasis remains fairly common in patients with this type of tumor [1,2], they might coexist with other primary tumors of variable histopathological composition confirming nonmetastatic origin. There seems to be a possible association of GISTs with other primary neoplasms. We present a case of a 75-year-old female who was diagnosed with a GIST neoplasm and was found to have an associated meningioma on incidental brain imaging. We highlight through our case the importance of a low threshold for further investigations of other potential neoplasms or associated syndromes in patients with GIST.

## Case presentation

**Figure 1:**
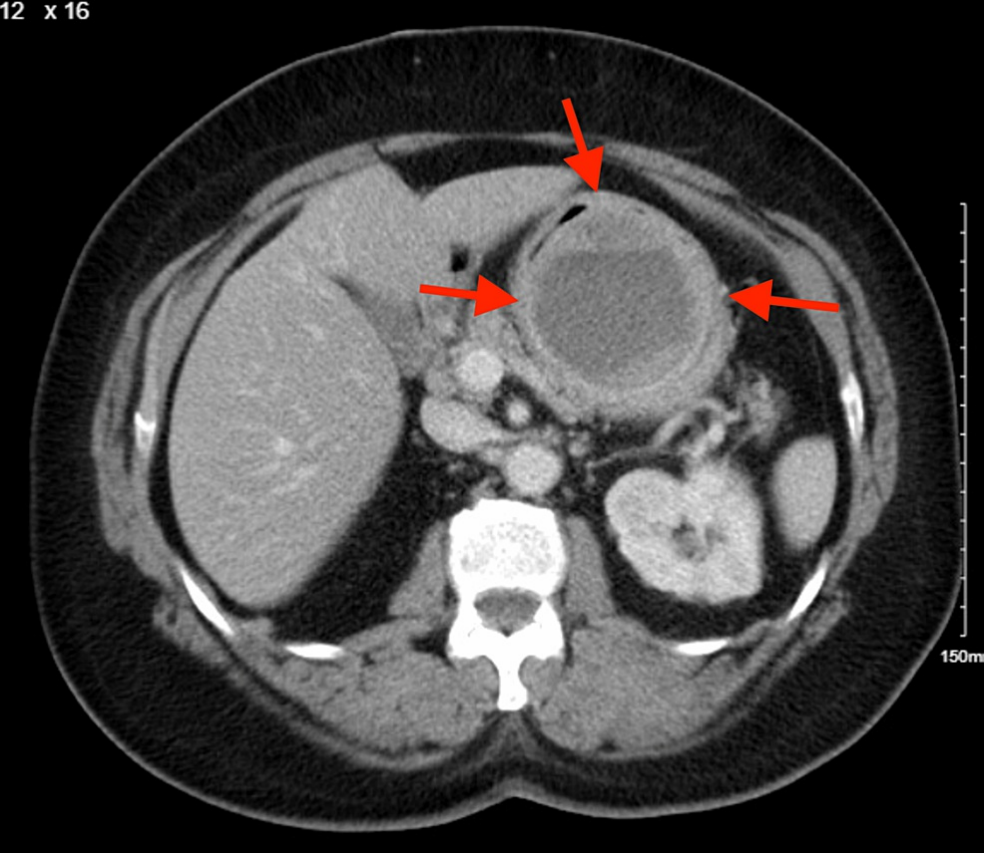
Mass representing a possible gastrointestinal stromal tumor The red arrows point to the mass.

**Figure 2:**
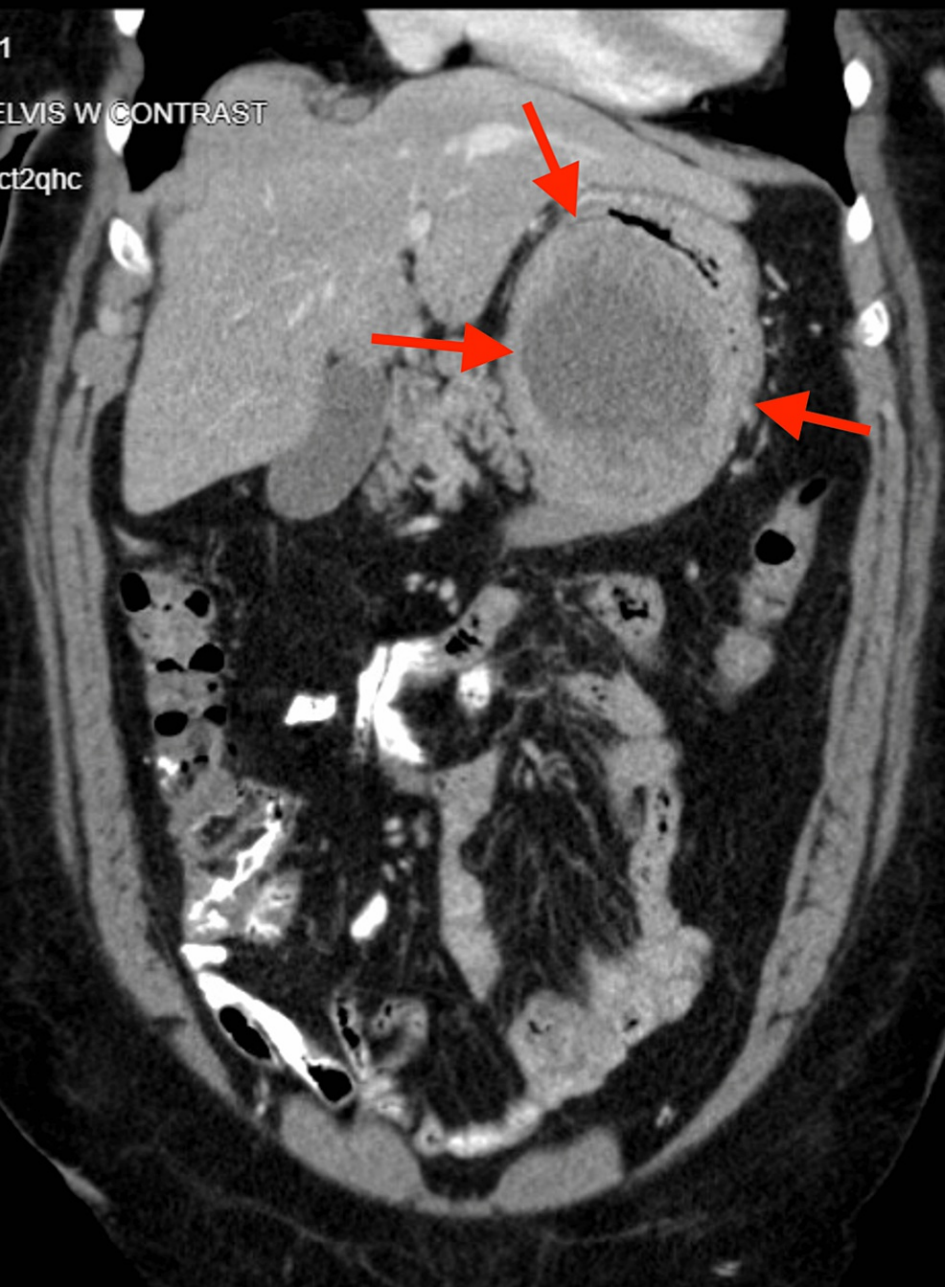
Mass representing a possible gastrointestinal stromal tumor The red arrows point to the mass.

A 75-year-old female with a past medical history of hypertension, hyperlipidemia, type II diabetes mellitus, gastroesophageal reflux disease, anemia, and osteoarthritis presented to the emergency room with several episodes of melena recurring daily for one week. The patient stated that she had similar random episodes a few months prior. Computed tomography (CT) of the chest, abdomen, and pelvis was obtained, revealing a centrally located necrotic gastric mass resembling the characteristics of a gastrointestinal stromal tumor (GIST) (Figure 1 and Figure 2).

The patient was admitted to the Medicine service for further workup. The Gastrointestinal (GI) service was consulted, and they performed an esophagogastroduodenoscopy (EGD) and colonoscopy with biopsies obtained from the mass. The specimens obtained revealed a large, oval-shaped mass with an ulcerated oozing surface and central necrosis. Pathology revealed gastric mucosa with associated small fragments of necrotic/hemorrhagic material with minute foci of viable spindle cells. The colonoscopy showed scattered polyps throughout the colon and grade II internal hemorrhoids. During the hospital stay, hemoglobin and hematocrit remained stable at 8.7 g/dL and 28.2%, respectively, the patient did not require a blood transfusion, and the melena resolved as well. A repeat EGD was required since biopsies were inconclusive. A fine needle aspiration revealed spindle cell neoplasms with confirmatory immunostains positive for cluster of differentiation 34 (CD34), CD117, and discovered on GIST-1 (DOG1) and negative for muscle creatine kinase (MCK), S100, and desmin, supporting the diagnosis of a GIST. The patient was discharged from the hospital and scheduled for a follow-up in a surgical oncology clinic.

**Figure 3:**
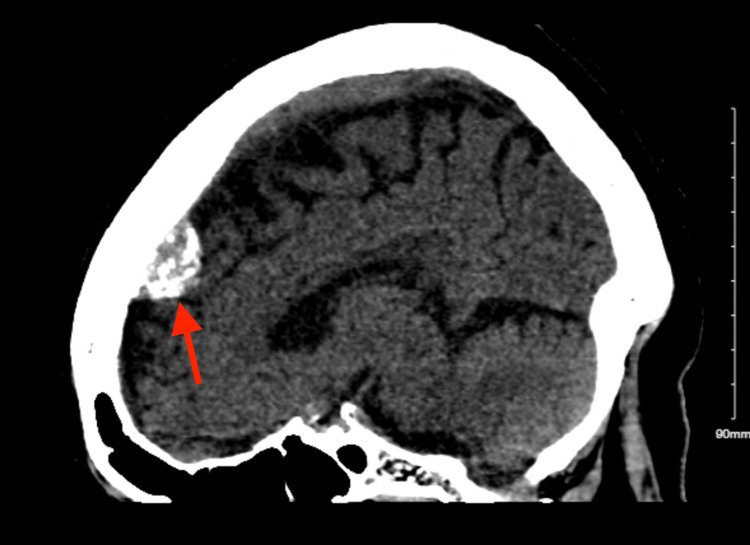
CT scan revealing a meningioma The red arrow points to the meningioma. CT: computed tomography

A few months later, the patient returned to the emergency room with dizziness, hazy vision, and a blood pressure of 226/118 mmHg. The patient denied any chest pain, dyspnea, or headaches. She stated that she took all her antihypertensive agents that morning but did not take her loop diuretic. CT scan of the head without contrast was performed and was unremarkable aside from a dural-based extra-axial hyperdense mass with calcification, which is highly suggestible of a meningioma (Figure 3).

The patient was admitted to the medical floor for treatment of a hypertensive emergency, without a clear etiology. Blood pressure medications were optimized, and the patient was ultimately discharged for outpatient follow-up. Ultimately, the GIST was excised, and the patient recovered without significant complications. The patient started on a tyrosine kinase inhibitor to prevent recurrence. After 10 years, the patient is still in remission.

## Discussion

Despite being a rare tumor [3], GIST represents the most common mesenchymal tumor of the gastrointestinal tract. The median age for incidents of GISTs is 65-69 years old, and it is very rare to be present in individuals under the age of 40. Additionally, there is a higher incidence of GISTs among individuals of Chinese descent [3]. Several different mutations are thought to be involved in the formation of a GIST and several immunohistochemical stains, which are useful in identifying a gist. The most common mutations in GISTs are in c-Kit (CD117) and platelet-derived growth factor alpha (PDGFA). Other immunohistochemical markers found in GISTs are DOG1, protein kinase C theta (PKC theta), CD34, alpha-smooth muscle actin (alpha-SMA), S100, and rarely desmin [3,4].

The vast majority of GISTs are caused by sporadic mutations; however, approximately 5% are related to genetic syndromes. These include primary familial GIST syndrome, neurofibromatosis type 1 (NF1), Carney-Stratakis syndrome, and the Carney triad. None of these syndromes however include meningiomas. Meningiomas are a key part of several syndromes, as well as neurofibromatosis type 2 (NF2) and schwannomatosis [5,6]. Additionally, patients with multiple endocrine neoplasia type 1 (MEN1) also have an increased risk of meningioma [7]. None of these syndromes include gastrointestinal stromal tumors. On the other hand, there has been a significant association between succinate dehydrogenase complexes and wild-type GIST. Such complexes have been most commonly associated with pheochromocytoma and paragangliomas alongside GIST. An association with meningioma has been described, but not with associated GIST tumors [8].

There does not appear to be a clear documented association between meningiomas and GIST occurring simultaneously in isolation as part of a syndrome. There are however reports of increased incidents of several other tumors in patients who have been diagnosed with GISTs [2]. Although a direct link between meningiomas and GISTs could not be clearly identified by these authors, the correlation remains a possibility. The abundant tumor markers that are associated with GISTs also lend credence to the fact that these other associated tumors stem from the same genetic mutation, thus resulting in a predisposition to such tumors.

## Conclusions

GISTs are rare tumors that can have significant effects on patients. It is well documented that GISTs are associated with other tumors. Although there is little literature on the mechanism of the associations between these tumors, there remains evidence to support correlation. Providers should have a very low threshold to perform further imaging for other possible tumors if symptoms arise in the indicated context of clinical management. Further research is required to elaborate on the potential genetic correlation between GIST tumors and meningiomas to determine whether this risk can be familial or coincidental.
